# Chemical Composition, Antibacterial and Antioxidant Activities of Six Essentials Oils from the *Alliaceae* Family

**DOI:** 10.3390/molecules191220034

**Published:** 2014-12-01

**Authors:** Dima Mnayer, Anne-Sylvie Fabiano-Tixier, Emmanuel Petitcolas, Tayssir Hamieh, Nancy Nehme, Christine Ferrant, Xavier Fernandez, Farid Chemat

**Affiliations:** 1 Université d’Avignon et des Pays du Vaucluse, UMR 408, INRA, GREEN extraction Team, Avignon 84000, France; E-Mails: d_mnayer@yahoo.fr (D.M.); anne-sylvie.fabiano@univ-avignon.fr (A.-S.F.-T.); emmanuel.petitcolas@univ-avignon.fr (E.P.); 2 Faculty of Agricultural Engineering and Veterinary Medicine, Lebanese University, Dekwaneh, Beirut 6573, Lebanon; E-Mails: tayssir.hamieh@ul.edu.lb (T.H.); nehmenancy@hotmail.com (N.N.); 3 Laboratory of Materials, Catalysis, Environment and Analytical Methods (MCEMA), Faculty of Sciences, Doctoral School of Sciences and Technology (EDST), Lebanese University, Beirut 6573-14, Lebanon; 4 Ferrant P.H.E, 2 Rue d’Ecottes, Rodelinghem 62610, France; E-Mail: ferrantphe@Wanadoo.fr; 5 Institut de Chimie de Nice, UMR 7272, Université de Nice-Sophia Antipolis/CNRS, Parc Valrose, Nice 06108, France; E-Mail: xavier.fernandez@unice.fr

**Keywords:** essential oils, *Allium* species, antioxidant activity, antibacterial activity

## Abstract

Six essential oils (EOs) from the *Alliaceae* family, namely garlic (*Allium sativum*), onion (*Allium cepa)*, leek (*Allium porrum)*, Chinese chive (*Allium tuberosum*), shallot (*Allium ascalonicum)* and chive (*Allium schoenoprasum)* were characterized by GC and GC-MS and evaluated for their functional food properties. Antibacterial properties were tested on five food-borne pathogens: Two Gram-positive *Staphylococcus aureus* (ATCC 25923), *Listeria monocytogenes (*ATCC 19115) and three Gram-negative *Salmonella* Typhimurium (ATCC 14028), *Escherichia coli* (ATCC 8739) and *Campylobacter jejuni* (ATCC 33291) bacteria. Antioxidant and radical-scavenging properties were tested by means of Folin-Ciocalteu and 2,2-diphenyl-1-picrylhydrazyl (DPPH) assays. Garlic, Chinese chive and onion EOs had the highest antibacterial activity whereas shallot and leek EOs were the strongest antioxidants. Heating caused a decrease in the antioxidant activity of these Eos, as shown in the Total Polar Materials (TPM) test. Suggestions on relationships between chemical composition and biological activities are presented. Results show that the EOs could be of value in the food industry as alternatives to synthetic antioxidants.

## 1. Introduction

*Allium* species, the most important genus of the *Alliaceae* family, are among the oldest cultivated vegetables. They have been used as ornamentals, spices, vegetables, or as medicines for curing various diseases. The *Allium* genus includes more than 700 species widely distributed all over the world [1] and appreciated due to their flavor, easy growth and long storage time. The species may differ in form and taste, but they are close in biochemical and phytochemical contents. *Allium* species are characterized by their rich content in sulfur compounds that are responsible for the organoleptic parameters [2,3] and contribute to the antioxidant and antimicrobial activities of these vegetables [4,5,6,7,8]. These volatile components also constitute the major part of the essential oils of these plants [9].

Garlic (*Allium sativum*) and onion (*Allium cepa*) are the most important *Allium* species consumed all over the world. The allicin derivative products (diallyl disulfide, diallyl trisulfide) found in garlic essential oils have shown good antimicrobial [10,11] and antioxidant activities [12]. The major compounds presented in onion EO are dipropyl disulfide and dipropyl trisulfide [10] and they have been reported to have antimicrobial and antioxidant activities [13]. Leek (*Allium porrum*), Chinese chive (*Allium tuberosum*), shallot (*Allium ascalonicum*) and chive (*Allium schoenoprasum*) are widely cultivated in Asian countries. They constitute important ingredients in many European and Asian diets [14] and they have been known for their medicinal properties [15,16,17,18,19,20]. The essential oils of these plants are believed to have antimicrobial and antioxidant activities, which are mostly associated with their sulfur compounds [21]. However, little attention has been paid to the compositions and properties of the essential oils of these *Allium* species.

Lipid oxidation and microbial alteration are the main reasons for the deterioration of quality, safety and shelf life of food. In fact, the presence of pathogens in food might be responsible for serious diseases leading to death. Gram-positive *Staphylococcus aureus*, *Listeria monocytogenes* and Gram-negative *Salmonella* Typhimurium, *Escherichia coli* and *Campylobacter jejuni* are among the most common bacteria present in food. *Staphylococcus aureus* is a pathogen of great importance in food processing. Some strains produce a poisonous toxin to humans which is resistant to high temperatures, gastric acidity and proteases; it can grow at high salt concentrations, low water activities and a relatively low pH. *Listeria monocytogenes* has the ability to grow at 4 °C; it can be present at high concentrations in cases of long storage. Its transmission might be maternal-fetal and through contaminated food. *Salmonella* Typhimurium is the major cause of food-borne diseases; it is a severe pathogen for humans associated with outbreaks caused mainly by contaminated meat and eggs. *Escherichia coli* is often used as an indicator of fecal contamination in food products; it constitutes about 80% of human intestinal flora. Finally, *Campylobacter jejuni* is the second most common cause of food poisoning after *Salmonella*; it has a high mobility which is important in the phenomenon of colonization in the intestinal tract.

Due to the increasing demand for natural ingredients, the use of essential oils for food preservation appears as a viable and healthy alternative to unpopular synthetic antioxidants. Therefore, this work was carried out to evaluate the chemical composition of six essential oils of garlic, onion, leek, Chinese chive, shallot and chive as well as their antioxidant and antimicrobial activities. Foremost, the extractions of EO were optimized using turbo hydrodistillation, then they were analyzed for their possible antioxidant activities by two methods, namely the Folin-Ciocalteu assay for the determination of the total phenol contents and the DPPH assay for their radical scavenging activity. Antibacterial tests using the disk diffusion method have been conducted against the five important food-borne pathogens mentioned above. In addition to that, the possible use of EOs in food processing under thermal conditions has been studied. The objective of this study was to determine the possibility of using the EOs as food additives for the food industry in order to satisfy the consumer demands by reducing the use of synthetic antioxidants.

## 2. Results and Discussion

### 2.1. Chemical Composition of the Essential Oils

Table 1 shows the chemical constituents, the relative percentage of the total chromatogram area according to the total compounds and the retention indexes of garlic, onion, leek, Chinese chive, shallot and chive essential oils.

GC-MS analysis of garlic EO identified 27 constituents representing more than 94.63% of the total EO. The major components were diallyl disulfide (37.90%), diallyl trisulfide (28.06%), allyl methyl trisulfide (7.26%), diallyl sulfide (6.59%), diallyl tetrasulfide (4.14%) and allyl methyl disulfide (3.69%). The profile obtained in this study was similar to that presented by Banerjee *et al.* [22] and Kim *et al.* [10]. Different studies on the composition of garlic essential oil show that diallyl disulfide and diallyl trisulfide are the two major compounds [4,23].

Thirty one constituents which represent more than 82.36% of the total EO were identified in the onion EO. The main components were dipropyl disulfide (30.92%), dipropyl trisulfide (17.10%), 1-propenyl propyl disulfide (7.26%) and methyl propyl trisulfide (5.20%). Dipropyl disulfide was reported by Corzomartinez *et al.* [4] to be the major compound present, which is in accordance with our results.

When leek EO was analyzed, 41 compounds which constitute more than 86.90% of the total EO were identified. Dipropyl disulfide was the major component, representing 47.70% followed by dipropyl trisulfide (15.01%), methyl propyl disulfide (4.48%), 1-propenyl propyl disulfide (3.75%) and methyl propyl trisulfide (3.19%). These results are in agreement with Casella *et al*. [23] who reported that dipropyl disulfide and dipropyl trisulfide were also the major compounds.

**Table 1:** Chemical composition of the essential oils of garlic, onion, leek, Chinese chive, shallot and chive.

Compounds	Essential Oils (% ± SD)							Identification Methods
	LRI_HP5_	Garlic	Onion	Leek	Chinese Chive	Shallot	Chive	
Isoamyl alcohol	762	-	0.13 ± 0.03	-	-	-	-	SM, LRI, Std
Methyl 1-propenyl sulfide ^a^	764	-	-	-	-	tr	-	SM, LRI
Dimethyl disulfide	767	1.12 ± 0.18	tr	0.28 ± 0.02	19.58 ± 2.62	tr	tr	SM, LRI, Std
(*Z*)-3-hexenal	769	0.15 ± 0.01	-	tr	tr	0.16 ± 0.03	tr	SM, LRI, Std
2-methylpentenal ^a^	776	0.15 ± 0.02	-	-	-	0.51 ± 0.07	-	SM, LRI
Hexanal	802	-	-	tr	tr	0.17 ± 0.02	-	SM, LRI, Std
Propanal diethyl acetal ^t^	813	-	-	tr	-	-	0.16 ± 0.03	SM
2-Ethylpyridine	836	0.10 ± 0.01	-	-	-	-	-	SM, LRI
(*E*)-hexenol	848	-	-	tr	-	-	-	SM, LRI, Std
1,3-Propanedithiol ^t^	851	-	-	tr	-	-	0.17 ± 0.01	SM
Diallyl sulfide	854	6.59 ± 0.55	tr	tr	1.47 ± 0.10	tr	tr	SM, LRI, Std
*n*-Hexanol	866	-	-	0.25 ± 0.02	-	-	-	SM, LRI, Std
Allyl propyl sulfide	867	0.09 ± 0.01	-	0.15 ± 0.01	-	0.16 ± 0.03	-	SM, LRI
Bis-(1-propenyl)-sulfide ^a^	884	0.08 ± 0.01	-	-	0.21 ± 0.01	-	-	SM, LRI
Dimethyl thiophene ^a^	871	-	-	-	-	-	0.15 ± 0.01	SM, LRI
Nonane	900	-	-	-	-	-	tr	SM, LRI, Std
Dimethyl thiophene ^a^	902	0.08 ± 0.01	0.18 ± 0.01	0.34 ± 0.03	-	0.21 ± 0.01	0.26 ± 0.01	SM, LRI
Allyl methyl disulfide	915	3.69 ± 0.02	-	-	14.37 ± 0.36	-	-	SM, LRI, Std
Methyl propyl disulfide	926	0.25 ± 0.01	2.11 ± 0.16	4.48 ± 0.33	1.21	3.26 ± 0.27	2.55 ± 0.13	SM, LRI
Methyl 1-propenyl disulfide ^a^	934	0.46 ± 0.02	0.51 ± 0.04	0.27 ± 0.03	6.07 ± 0.13	1.33 ± 0.13	0.62 ± 0.03	SM, LRI
Dimethyl trisulfide	962	0.33 ± 0.01	0.30 ± 0.01	0.12 ± 0.01	14.34 ± 0.05	1.22 ± 0.06	0.65 ± 0.02	SM, LRI, Std
2-Pentylfuran	990	-	-	-	-	-	0.14 ± 0.01	SM, LRI
Diallyl disulfide	1084	37.90 ± 0.07	-	-	5.14 ± 0.21	0.13	-	SM, LRI, Std
Allyl propyl disulfide	1088	-	0.42 ± 0.08	0.73 ± 0.04	-	0.55 ± 0.06	0.44 ± 0.02	SM, LRI
Linalool	1101	-	-	-	1.75 ± 0.14	-	-	SM, LRI, Std
Dipropyl disulfide	1105	0.25 ± 0.06	30.92 ± 0.03	47.70 ± 0.03	1.24 ± 0.05	15.17 ± 0.18	19.49 ± 0.08	SM, LRI
Ethyl-3-(methylthio)propionate	1113	0.09 ± 0.01	-	-	-	-	-	SM, LRI
1-Propenyl propyl disulfide ^a^	1116	-	7.26 ± 0.06	3.75 ± 0.02	-	4.57 ± 0.05	5.84 ± 0.05	SM, LRI
2,4,5-tTrithiahexane ^t^	1118	-	-	-	0.15 ± 0.01	-	-	SM
3,5-Dimethyl-1,2,4-trithiolane	1126	-	0.12 ± 0.01	0.21 ± 0.01	-	-	-	SM, LRI
Allyl methyl trisulfide	1131	7.26 ± 0.05	-	-	7.24 ± 0.38	-	0.2	SM, LRI
Menthone	1145	-	-	-	1.91 ± 0.12	-	-	SM, LRI, Std
Methyl propyl trisulfide	1148	-	5.20 ± 0.02	3.19 ± 0.02	-	9.20 ± 0.10	8.47 ± 0.10	SM, LRI
Methyl 1-propenyl trisulfide	1153	-	0.34 ± 0.01	0.13 ± 0.01	-	0.50 ± 0.08	0.36 ± 0.01	SM, LRI
Methyl-1-(methylthio)ethyl-disulfide ^t^	1159		0.47 ± 0.05	0.15 ± 0.01	-	0.68 ± 0.05	0.51 ± 0.02	SM
*n*-Nonanol	1172		-	0.47 ± 0.01	-	-	0.15 ± 0.01	SM, LRI, Std
3,4-Dihydro-3-vinyl-1,2-dithiin ^t^	1177	0.13 ± 0.02	-	-	-	-	-	SM
Borneol	1183	-	-	-	-	0.33 ± 0.01	0.85 ± 0.01	SM, LRI, Std
α-Terpineol	1187	-	-	-	0.32 ± 0.01	-	-	SM, LRI, Std
Methyl salicylate	1189	-	-	-	0.46 ± 0.01	-	-	SM, LRI, Std
Methyl chavicol	1196	-	-	-	-	0.11 ± 0.01	-	SM, LRI, Std
Dimethyl tetrasulfide	1206	0.56 ± 0.01	0.15 ± 0.01	-	2.82 ± 0.19	0.46 ± 0.01	0.39 ± 0.01	SM, LRI, Std
3-Ethyl-5-methyl-1,2,4-trithiolane ^a, t^	1216	-	0.13 ± 0.01	-	-	0.11 ± 0.01	-	SM
3-Ethyl-5-methyl-1,2,4-trithiolane ^a, t^	1219	-	0.16 ± 0.01	0.54 ± 0.01	-	0.17 ± 0.01	-	SM
Butyl thiocyante	1239	-	-	-	-	0.21 ± 0.01	-	SM, LRI
Methyl 1-(methylthiopropyl) disulfide	1249	-	0.36 ± 0.04	0.12 ± 0.01	0.59 ± 0.04	0.52 ± 0.06	0.20 ± 0.01	SM, LRI
2-Undecanone	1292	-	0.53 ± 0.01	-	0.62 ± 0.04	0.82 ± 0.01	0.10 ± 0.01	SM, LRI
Tridecane	1301	-	0.49 ± 0.05	-	-	-	-	SM, LRI, Std
Diallyl trisulfide	1305	28.06 ± 0.63	-	-	-	-	-	SM, LRI
3-Methoxyoctane ^t^	1311	1.10 ± 0.03	-	-	-	0.88 ± 0.01	1.24 ± 0.02	SM
Dipropyl trisulfide	1328	tr	17.10 ± 0.28	15.01 ± 0.27	tr	11.14 ± 0.14	15.21 ± 0.18	SM, LRI
1-Propenyl propyl trisulfide ^a^	1332	-	-	0.43 ± 0.02	-	1.36 ± 0.02	-	SM, LRI
Allyl propyl trisulfide	1334	-	1.84 ± 0.01	1.58 ± 0.03	-	1.97 ± 0.01	1.92 ± 0.02	SM, LRI
Di-1-propenyl trisulfide	1347	0.23 ± 0.05	3.07 ± 0.01	-	-	0.26 ± 0.02	0.14 ± 0.01	SM, LRI
Eugenol	1356	0.21 ± 0.01	-	-	0.26 0.01	-	-	SM, LRI
Benzyl thiocyanate	1359	-	-	0.20 ± 0.02	0.27 ± 0.02	-	-	SM, LRI, Std
α-Copaene	1370	-	-	-	-	-	0.14 ± 0.01	SM, LRI, Std
Allyl methyl tetrasulfide	1371	1.07 ± 0.03	-	-	1.21 ± 0.09	-	-	SM, LRI
Benzyl methyl disulfide ^t^	1373	-	-	-	0.37 ± 0.04	-	-	SM
Geranyl acetate	1386	-	-	0.83 ± 0.01	-	-	-	SM, LRI
Methyl eugenol	1405	-	-	-	-	0.14 ± 0.03	-	SM, LRI, Std
6,10-Dimethyl 2-undecanone	1406	-	-	-	-	-	0.32 ± 0.01	SM, LRI
β-Caryophyllene	1410	-	-	-	-	-	0.17 ± 0.01	SM, LRI, Std
3,6-Dimethyl-2,4,5,7 tetrathioctane ^t^	1423	-	-	0.39 ± 0.04	-	-	0.11 ± 0.01	SM
2-Hexyl-5-methyl 3(2*H*)-furanone	1440	-	1.26 ± 0.01	0.13 ± 0.01	-	5.40 ± 0.15	0.15 ± 0.01	SM, LRI
β-Selinene	1445	-	-	-	-	-	0.25 ± 0.03	SM, LRI
(*E*)-β-Farnesene	1457	-	-	-	-	-	1.50 ± 0.06	SM, LRI
β-Ionone	1481	-	-	-	0.13 ± 0.01	-	0.30 ± 0.04	SM, LRI, Std
Methyl-1-propylthioethyl tetrasulfide	1487	-	-	0.43 ± 0.01	-	-	-	SM, LRI
2-Tridecanone	1496	-	0.32 ± 0.03	-	-	0.63 ± 0.01	-	SM, LRI
γ-Cadinene	1506	0.10 ± 0.01	-	-	-	-	-	SM, LRI
α-Farnesene	1509	-	-	-	-	-	2.56 ± 0.08	SM, LRI
β-Sesquiphellandrene	1521	-	-	-	-	-	0.15 ± 0.01	SM, LRI
Diallyl tetrasulfide	1538	4.14 ± 0.11	-	-	0.22 ± 0.02	-	-	SM, LRI, Std
2-Methyl-3,4-dithiaheptane	1558	-	6.48 ± 0.08	2.03 ± 0.04	-	4.42 ± 0.05	3.70 ± 0.05	SM, LRI
Dipropyl tetrasulfide	1573	-	0.55 ± 0.04	-	-	-	0.35 ± 0.01	SM, LRI
Tetradecanal	1607	-	-	0.15 ± 0.02	-	-	-	SM, LRI
Dimethyl pentasulfide	1686	-	-	-	0.23 ± 0.02	-	-	SM, LRI
Propyl-1-(propylthio)ethyl trisulfide ^t^	1700	-	-	0.28 ± 0.01	-	0.44 ± 0.03	1.12 ±0.02	SM
6,10,14-Trimethyl-2-pentadecanone	1845	-	-	0.52 ± 0.01	0.16 ± 0.01	0.23 ± 0.01	1.35 ± 0.01	SM, LRI
Benzyl salicylate	1857	-	-	-	-	0.27 ± 0.01	-	SM, LRI
2,4-Diméthyl-5,6-dithia-2,7-nonadienal	1885	0.44 ± 0.02	-	-	-	-	-	SM, LRI
Methyl palmitate	1928	-	0.81 ± 0.04	0.17 ± 0.01	0.15 ± 0.01	1.39 ± 0.02	0.30 ± 0.01	SM, LRI, Std
Palmitic acid	1970	-	-	0.76 ± 0.01	1.58 ± 0.10	-	2,17 ± 0.06	SM, LRI, Std
Ethyl palmitate	1996	-	0.42 ± 0.02	0.48 ± 0.01	0.41 ± 0.03	0.43 ± 0.01	0.56 ± 0.01	SM, LRI, Std
Methyl linoleate	2093	-	0.55 ± 0.01	-	0.14 ± 0.01	-	0.25 ± 0.01	SM, LRI, Std
Methyl linolenate	2099	-	-	-	0.16 ± 0.01	-	-	SM, LRI, Std
Phytol	2113	-	-	0.16 ± 0.01	0.61 ± 0.03	-	-	SM, LRI
Ethyl linoleate	2161	-	-	0.26 ± 0.01	0.21 ± 0.01	0.42 ± 0.04	0.35 ± 0.01	SM, LRI, Std
Ethyl oleate	2167	-	0.18 ± 0.01	-	-	0.36 ± 0.01	-	SM, LRI, Std
Ethyl linolenate	2168	-	-	0.21 ± 0.01	-	-	0.35 ± 0.02	SM, LRI
Ethyl α-linolénate	2244	-	-	-	0.19 ± 0.02	-	-	SM, LRI

SD = Standard Deviation. Compositional values less than 0.1% are denoted as traces (tr). Presence of a compound is indicated by its GC-FID percentage with SD, absence is indicated by “-”. ^a^: Correct isomer not identified. ^t^: Tentative.

In the Chinese chive EO, 37 compounds were identified, representing 85.79% of the total oil. The EO consisted mainly of dimethyl disulfide (19.58%), allyl methyl disulfide (14.37%), dimethyl trisulfide (14.34%), allyl methyl trisulfide (7.24%), methyl 1-propenyl disulfide (6.07%) and diallyl disulfide (5.14%).

Concerning the shallot EO, 42 compounds which represent more than 70.29% of the total EO were identified. The major components were dipropyl disulfide (15.17%), dipropyl trisulfide (11.14%), methyl propyl trisulfide (9.20%), 1-propenyl propyl disulfide (4.57%) and methyl propyl disulfide (3.26%).

GC-MS analysis of chive EO identified 48 components, representing more than 76.36% of the total EO. The main compounds found in chive EO were dipropyl disulfide (19.49%), dipropyl trisulfide (15.21%), methyl propyl trisulfide (8.47%) and 1-propenyl propyl disulfide (5.84%).

**Figure 1:**
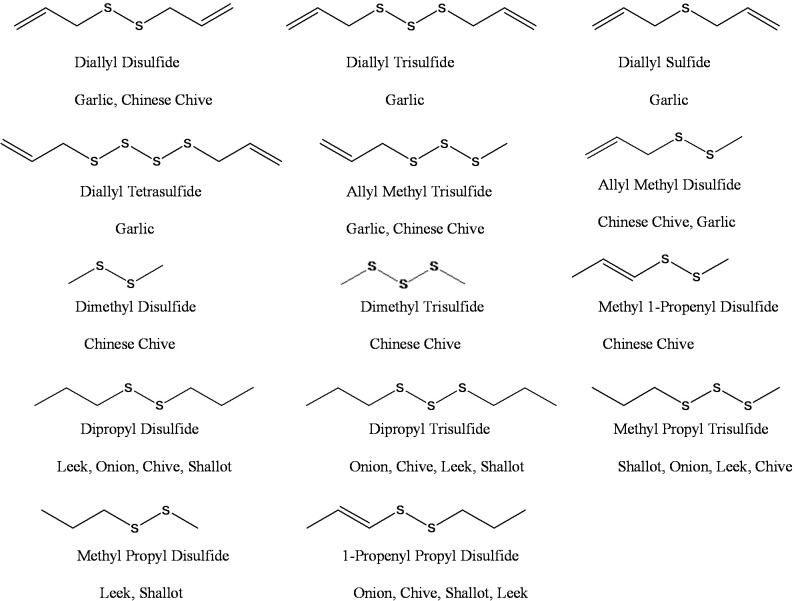
Structure of major compounds of the essential oils of garlic, onion, leek, Chinese chive, shallot and chive.

According to these results, diallyl disulfide was mainly present in garlic and Chinese chive EO whereas dipropyl disulfide and dipropyl trisulfide were the most representative compounds found in the essential oils of onion, leek, shallot and chive. The structures of the major bioactive compounds are presented in Figure 1.

### 2.2. In Vitro Antimicrobial Activity

The *in vitro* antibacterial activity of the six essential oils against the tested microorganisms (Gram-positive and Gram-negative bacteria) was assessed by the disc diffusion method by measuring the inhibition zones. According to the results presented in Table 2, the essential oils with the highest antibacterial effects produced inhibition zones larger than 20 mm diameter.

**Table 2:** Antibacterial activity of garlic, onion, leek, Chinese chive, shallot, chive EO and positive control after 48 h ^1^.

Pathogens	Inhibition Diameter (mm) ^2^ Including Disk Diameter of 6.0 mm			
	Garlic	Onion	Leek	
*Staphylococus aureus*	20.0 ± 0.0 ^a^	15.5 ± 2.1 ^a^	10.0 ± 0.0 ^a^	
*Salmonella* Typhimurium	9.0 ± 1.0 ^c^	12.0 ± 1.8 ^a b^	6 mm	
*Listeria monocytogenes*	23.0 ± 1.4 ^a^	15.0 ± 1.4 ^a^	6 mm	
*Escherichia coli*	9.3 ± 0.9 ^c^	6 mm	6 mm	
*Campylobacter jejuni*	12.6 ± 2.1 ^b^	9.0 ± 1.2 ^b^	9.3 ± 1.9 ^a^	
**Pathogens**	**Inhibition Diameter (mm) ^2^ Including Disk Diameter of 6.0 mm**			
	**Chinese Chive**	**Shallot**	**Chive**	**Positive Control**
*Staphylococus aureus*	18.5 ± 0.7 ^a^	20.0 ± 0.1 ^a^	11.5 ± 0.7 ^a^	30.6 ± 0.6 ^a^
*Salmonella* Typhimurium	9.3 ± 1.2 ^b^	11.3 ± 2.3 ^b^	6 mm	25.6 ± 1.3 ^b^
*Listeria monocytogenes*	6 mm	6 mm	6 mm	28.0 ± 0.5 ^a^^ b^
*Escherichia coli*	9.0 ± 1.4 ^b^	6 mm	6 mm	25.5 ± 1.1 ^b^
*Campylobacter jejuni*	21.0 ± 1.7 ^a^	11.6 ±1.5 ^b^	±2.1 ^a^	25.3 ± 1.2 ^b^

^1^ Results are analyzed according to Ponce *et al.*, [24]; ^2^ Results are mean ± SD values of three replications. For the same essential oil, values followed by different letters within the same column are significantly different (*p* < 0.05) according to Tukey’s HSD test.

The positive control amoxicillin/clavulanic acid was extremely effective on all tested bacteria, with inhibition zones ranging from 25.3 to 30.6 mm. *Staphylococcus aureus* was highly sensitive to the control (30.6 mm, *p* < 0.05), followed by *Listeria monocytogenes* (28.0 mm). The three Gram-negative bacteria were also highly sensitive with no statistical difference between them (25.6 mm for *Salmonella* Typhimurium, 25.5 mm for *Escherichia coli* and 25.3 mm for *Campylobacter jejuni*).

Among the essential oils, the most effective in this respect was garlic oil inhibiting all five bacteria tested with different sensitivities. Garlic EO was highly effective (*p* < 0.05) on *Staphylococcus aureus* and *Listeria monocytogenes*, showing inhibitory zones of 20.0 and 23.0 mm, respectively. Garlic oil exhibited lower inhibition activity against *Campylobacter jejuni*, *Escherichia coli* and *Salmonella* Typhimurium with diameter inhibition halos of 12.6, 9.3 and 9.0 mm, respectively.

The two second most effective EOs were Chinese chive and onion oils. They inhibited four bacteria. *Campylobacter jejuni* and *Staphylococcus aureus* were highly sensitive (*p* < 0.05) to Chinese chive EO with inhibition zones of 21.0 and 18.5 mm, respectively. *Salmonella* Typhimurium and *Escherichia coli* were sensitive to Chinese chive EO (9.3 and 9.0 mm, respectively) whereas *Listeria monocytogenes* was resistant to the same EO. *Staphylococcus aureus* and *Listeria monocytogenes* were highly sensitive (*p* < 0.05) to onion oil with diameters of 15.5 and 15.0 mm, respectively. *Salmonella* Typhimurium and *Campylobacter jejuni* were also sensitive with inhibition zones of 12.0 and 9.0 mm, respectively. *Escherichia coli* was the only resistant bacteria to this essential oil.

Shallot essential oil inhibited three of the five bacteria tested; it was extremely effective (*p* < 0.05) on *Staphylococcus aureus* (20.0 mm) and active on *Campylobacter jejuni* (11.6 mm) and *Salmonella* Typhimurium (11.3 mm).

Leek and chive essential oils were both active on two bacteria, with inhibition zones for *Staphylococcus aureus* of 10.0 and 11.5 mm, respectively, and for *Campylobacter jejuni* of 9.3 and 10.3 mm respectively. The results were in a accordance with the ones already published showing that garlic, onion, leek, Chinese chive, shallot and chive EO had antibacterial activity against Gram-negative and Gram-positive bacteria [10,14,21,23,25,26,27,28].

Studies show that diallyl sulfide exerts good antimicrobial activity [4,7,29,30]. In fact, in sulfide compounds, a greater number of sulfur atoms is found to result in stronger antimicrobial activity [10,11,14]. This explains the good antimicrobial effect of garlic and Chinese chive EO which have diallyl disulfide in their compositions (37.90% and 5.14% respectively). Moreover, garlic EO also contains diallyl trisulfide (28.06%) and diallyl tetrasulfide (4.14%). Thus, the richness in sulfur atoms may have contributed in the effectiveness of the EO activity. The high antimicrobial activity of Chinese chive EO may also be attributed to other sulfide compounds such as dimethyl disulfide (19.58%), allyl methyl disulfide (14.37%), dimethyl trisulfide (14.34%) and allyl methyl trisulfide (7.24%).

Studies on the antimicrobial activity of dipropyl disulfide and dipropyl trisulfide, which are the main components in onion, leek, shallot and chive EO, are scarce. However, it was noted that dipropyl trisulfide demonstrated antimicrobial activity against *Staphylococcus aureus* [10]. The antibacterial activity of these EO may be related to the propyl derivatives.

Several studies report that EO act more on Gram-positive than Gram-negative bacteria [31]; this is due to the difference in cell wall composition [32]. However, there is no general rule with respect to the Gram sensitivity because many controversies exist in the various published works. Thus the Gram-negative bacteria *Campylobacter jejuni* has been described as particularly sensitive to the action of EO [33]. This is in agreement with our results since Gram-negative *Campylobacter jejuni* showed sensitivity to the six essential oils. Among the Gram-positive bacteria, *Staphylococcus aureus* was sensitive to all EOs.

Due to the complexity of the chemical composition of the essential oils, their mechanisms of action have not been clarified yet. However, assumptions on their activity for targeting different bacterial structures might be proposed. Their hydrophobicity allows them to attack the phospholipid membrane of the cell and to increase its permeability. Therefore, the contents of the cells are lost leading to bacterial death [34]. More specifically, the relation between chemical structure and antibacterial activities of sulfides is not fully understood. However, Kyung [35] reported that the sulfides can damage the microbial cells by reacting with SH groups of cellular proteins to generate mixed disulfides.

### 2.3. Total Phenolic Content

The Total Phenolic Contents (TPCs) of the garlic, onion, leek, chive, shallot and Chinese chive essential oils are presented in Table 3. The positive control BHT had the highest phenol content (*p* < 0.05, 46.77 mg GAE/g). Among the six essential oils, shallot and leek oils showed the highest TPCs (*p* < 0.05, 1.14 and 10.79 mg GAE/g, respectively). Chive and garlic EO had lower TPCs (6.76 and 5.61 mg GAE/g, respectively) whereas Chinese chive and onion EO showed the lowest amount of TPCs (4.24 and 3.29 mg GAE/g respectively) with no statistical difference between them. Data on the TPCs of these essential oils is not available. However, Lu *et al.*, and Yang *et al.* [36,37] showed that shallot extracts had the highest phenol content (17.18 mg GAE/g fresh weight and 114.70 mg GAE/100 g sample, respectively) among different onion varieties. Leek extracts were also reported to be rich in phenols [16,19]. Phenolic contents are good indicators of antioxidant activity, their high redox potential enables them to act as hydrogen donors or radical scavengers [38,39]. They are also reported to play an important role in the antimicrobial potential of the EO [40,41,42].

**Table 3:** Total Phenol contents (TPCs) of garlic, onion, leek, Chinese chive, shallot, chive EO and BHT.

Essential Oils	Total Phenol Contents GAE * (mg/g)
Garlic	5.61 ± 0.69 ^c^
Onion	3.29 ± 0.12 ^d^
Leek	10.79 ± 0.53 ^b^
Chinese chive	4.24 ± 0.11 ^d^
Shallot	11.14 ± 0.43 ^b^
Chive	6.76 ± 0.37 ^c^
BHT	46.77 ± 0.81 ^a^

*: GAE: Gallic acid equivalent (mg/g). Values followed by the same letter within the same column are not significantly different (*p* > 0.05) according to Tukey’s HSD test.

### 2.4. DPPH Radical Scavenging Activity

The stable free radical DPPH was used to test the ability of the essential oils and BHT standard to donate the hydrogen atom. Table 4 shows the percentage of DPPH inhibition by the different concentrations of each EO and BHT. A concentration-dependent scavenging activity was found for the studied EO. All essential oils were able to reduce the stable free radical 2,2-diphenyl-1-picrylhydrazyl (DPPH) to the yellow diphenylpicrylhydrazine with varying degrees of scavenging capacities. Great bleaching action (from purple to yellow) reflected a higher antioxidant activity and thus a lower IC_50_ (Table 4). The values of IC_50_ were in the following order: BHT ˂ shallot ˂ leek ˂ chive ˂ garlic ˂ Chinese chive ˂ onion. Positive control BHT was the strongest antioxidant with IC_50_ value of 0.03 mg/mL. Among essential oils, shallot and leek EO showed the strongest radical scavenging effect with IC_50_ values of 2.70 and 4.49 mg/mL, respectively. This activity was followed by chive (5.59 mg/mL) and garlic EO (7.67 mg/mL). Chinese chive and onion EO showed the lowest scavenging activity with IC_50_ values of 12.16 and 20.19 mg/mL, respectively.

The antioxidant activity of these plants is attributed partly to its sulfur compounds, which represent the main constituents of these essential oils [4,7,43]. Amagase *et al.* [12] reported that diallyl polysulphides contributed to the antioxidant properties of the essential oils.

**Table 4:** Antioxidant activity of garlic, onion, leek, Chinese chive, shallot, chive EO and BHT at different concentrations measured by DPPH method.

Essential Oils	DPPH Inhibition (%)						
	2 mg/mL	4 mg/mL	8 mg/mL	12 mg/mL	16 mg/mL	20 mg/mL	IC_50_ *
Garlic	31.35 ± 1.91	37.93 ± 1.59	51.07 ± 0.67	64.22 ± 0.78	77.37 ± 0.79	90.52 ± 0.59	7.67
Onion	22.30 ± 0.97	25.34 ± 0.19	31.43 ± 1.60	37.52 ± 0.32	43.61 ± 0.42	49.70 ± 1.49	20.19
Chinese chive	17.93 ± 0.89	24.24 ± 1.39	36.85 ± 1.38	49.46 ± 0.30	62.08 ± 0.68	74.69 ± 0.31	12.16
Chive	39.05 ± 0.33	45.15 ± 0.11	57.34 ± 1.14	69.54 ± 1.45	81.73 ± 0.11	93.93 ± 1.70	5.59
	**1 mg/mL**	**2 mg/mL**	**3 mg/mL**	**4 mg/mL**	**5 mg/mL**		
Leek	9.90 ± 1.32	21.37 ± 1.70	32.84 ± 1.46	44.31 ± 0.64	55.78 ± 1.49		4.49
Shallot	29.95 ± 1.18	42.59 ± 1.11	51.99 ± 1.44	61.38 ± 1.58	70.77 ± 1.64		2.70
	**0.01 mg/mL**	**0.02 mg/mL**	**0.04 mg/mL**	**0.06 mg/mL**	**0.08 mg/mL**		
BHT	16.01 ± 1.71	35.3 ± 1.27	68.93 ± 2.01	85.50 ± 0.13	91.21 ± 0.41		0.03

* IC_50_: concentration (mg/mL) for a 50% inhibition.

The different antioxidant activities between these essential oils may be due to the variability of the composition and concentration of sulfides. It may also be attributed to the presence and synergy of different minor compounds. It is interesting to note that a good correlation between DPPH and Folin-Ciocalteu test was found, which is in the same order for all essentials oils. Both tests confirm that shallot exerts the strongest antioxidant activity and onion the lowest. This result indicates that the phenolic compounds of *Allium* species contribute to their antioxidant properties.

### 2.5. Heating Test

All samples (sunflower oil used as control and its mixture with each EO) display similar TPM results since the total heating times required to reach the maximal TPM value of 25% were quite close (ranging from 12 to 15 h). This might be attributed to the thermal degradation of sulfur and phenolic compounds present in the EO. These findings are in agreement with Wangcharoen and Morasuk [44] who reported that heat treatment caused the degradation of these constituents.

## 3. Experimental Section

### 3.1. Plant Materials, Chemicals and Standards

The bulbs of garlic, onion, leek, Chinese chive, shallots and chive were purchased from a local supermarket in Avignon province (France). Glycerol and Butylated Hydroxytoluene (BHT) were purchased from Sigma Aldrich (St. Louis, MO, USA), Trypticase Soy Agar was obtained from Biomerieux (Marcy-l’Étoile, France). Nutrient Broth and Mueller Hinton Agar were bought from VWR (London, UK), Nutrient Agar and Amoxicillin/Clavulanic acid were purchased from Himedia (Mumbai, India), Peptone water was obtained from SRL (Mumbai, India). Methanol was supplied by Fisher Scientific (Loughborough, UK). Folin-Ciocalteu reagent and Gallic acid were obtained from Isitec Lab-Seppal (Montauban, France) and DPPH was supplied by Sigma Aldrich (Munich, Germany).

### 3.2. Essential Oils: Extraction and Yield

**Figure 2:**
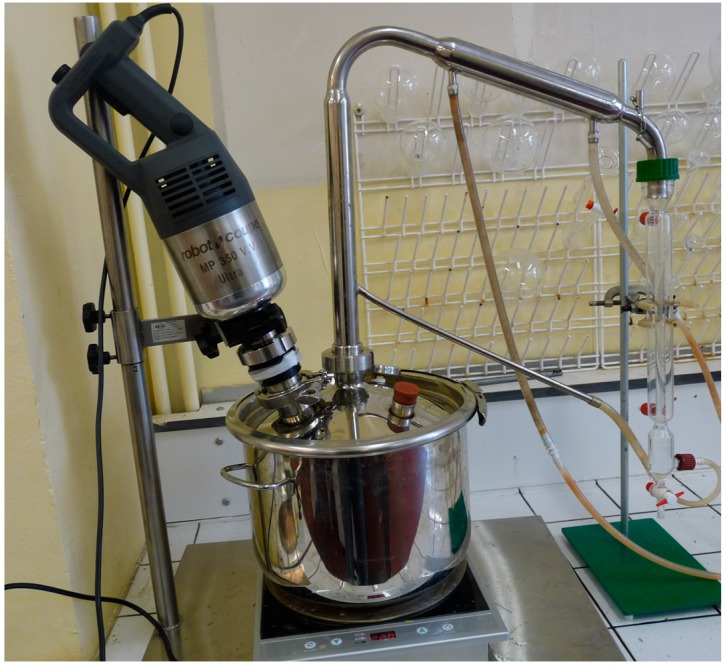
Extraction of essential oils by turbo hydrodistillation.

The EO were extracted by turbo hydrodistillation (Figure 2). This technique is similar to that of the conventional one. The difference lies in the installation of a stainless steel stirrer in the apparatus. It is equipped with blades to cut the bulbs into small pieces during distillation to increase and speed up the extraction process. Five kilograms of the different fresh *Allium* vegetables were soaked in distilled water (8000 mL) and submitted to turbo hydrodistillation with a Clevenger-type apparatus. The studied EOs, which have a higher density than water, sank to the bottom while water floated to the top. An amount of the EOs was lost due to their backflow into the apparatus. The EO extractions were optimized by adding petroleum ether to the system. This solvent trapped the essential oils at the top avoiding their decantation and consequently their loss. Then, the EOs were separated and filtered over sodium sulfate to eliminate all traces of water. The petroleum ether was evaporated in a rotary evaporator at 40 °C. The EOs were collected and stored at 4 °C away from light until use. The yields of the essential oils per 1 ton (g/t) of raw material were: 1300 to 2000 g/t for garlic, 60 to 130 g/t for onion, 80 to 110 g/t for leek, 200 to 300 g/t for Chinese chive, 80 to 120 g/t for shallot and 30 to 40 g/t for chive.

### 3.3. GC-FID and GC-MS Identification

The essential oils were analyzed using gas chromatography-mass spectrometry (GC-MS) to identify their chemical constituents. Essential oils were analyzed first by gas chromatography coupled to flame ionization detector (FID). These analyses were performed by using a 7890A Gas Chromatograph (Agilent, Massy, France) with a non-polar HP-5MS column (5% phenyl 95% methylsiloxane, 30 m × 0.25 mm × 0.25 µm) (Agilent). Injection of 0.4 µL samples was carried out with a split ratio 1:100, the carrier gas was H_2,_ and injection temperature was 250 °C. The oven temperature range progressed from 40 to 250 °C at 2 °C·min^−1^ (for 60 min). Then, gas chromatography coupled with mass-spectra were performed on a 5975C mass spectrometry detector (Agilent), using the same column as GC-FID. GC-MS spectra were obtained using the following conditions: the ratio split was 1:100, injection volume was 0.4 µL and the carrier gas was helium. Injection temperature was 250 °C and the oven temperature range progressed from 40 to 250 °C at 2 °C·min^−1^ (for 60 min). The ionization mode used was electronic impact at 70 eV and the mass range between 35 and 400 was scanned. Essential oils were identified by comparison of their GC linear retention indices (RI), determined with reference to a homologous series of alkanes. Identification was confirmed by comparison of their spectral mass with authentic samples, with those stored in the MS database (home-made and commercial libraries: Wiley 6N, NIST 98) and with index retention literature data [45] using the Automatic Mass Spectral Deconvolution and Identification System (AMDIS) software. Three repeated injections were performed for the quantitative analysis of the constituents.

### 3.4. Bacterial Strains

Two Gram-positive bacteria *Staphylococcus aureus (*ATCC 25923), *Listeria monocytogenes* (ATCC 19115) and two Gram-negative bacteria *Salmonella* Typhimurium (ATCC 14028), *Escherichia coli* (ATCC 8739) were supplied by the Lebanese Agricultural Research Institute (LARI, Fanar, Lebanon). Gram-negative bacteria *Campylobacter jejuni* (ATCC 33291) was supplied by the Lebanese University, Faculty of Agricultural Engineering and Veterinary Medicine.

Bacterial cultures were frozen at −20 °C in Nutrient Broth containing 20% glycerol (v/v). Throughout the experiments, the strains were subcultured every month on Trypticase Soy Agar and kept at 4 °C. Before use, bacteria were activated in Nutrient Broth and incubated for 24 h at 42 °C for *Campylobacter jejuni* under microaerophilic conditions and 24 h at 37 °C for the other strains. The bacterial suspension was then diluted in Peptone water to provide initial cell counts of about 10^6^ CFU/mL.

### 3.5. Screening for Antibacterial Activity

The paper disc diffusion method was applied to determine the antibacterial activity of the essential oils. One mL of the suspension of the tested microorganism (10^6^ CFU/mL) was spread on plates containing 20 mL Muller Hinton Agar. Filter paper discs of 6.0 mm diameter (Whatman n° 40) were individually impregnated with 15 µL of essential oil, then laid on to the surface of the inoculated plates. A disc containing 30 μg amoxicillin/clavulanic acid was placed in the plate as a positive control. At the end of incubation time (48 h at 42 °C for *Campylobacter jejuni* under microaerophilic conditions and 48 h at 37 °C for the other bacteria), positive antibacterial activities were established by the presence of measurable inhibition zones. The antimicrobial activity was recorded as the width (in millimetres, diameter of the disc included) of the inhibition zones after incubation using a ruler. This sensitivity was classified according to Ponce *et al.* [24] as follows: not sensitive for diameter less than 8 mm; sensitive for diameter of 9–14 mm; very sensitive for diameter of 15–19 mm and extremely sensitive for diameter larger than 20 mm. Each test was performed in three replicates.

### 3.6. Folin-Ciocalteu Assay

The Total Phenol Contents (TPCs) was determined using Folin–Ciocalteu reagent following the procedure described by the supplier of the kit [46]. A methanolic solution of essential oil (EO; 20 mg/mL, 100 µL) was introduced into test tubes followed by Folin-Ciocalteu reagent (2 mL) and alkaline buffer (1 mL). The tubes were vortexed and allowed to stand for 1 h in the dark. Absorption at 760 nm was measured with a 8453 diode-array spectrophotometer (Hewlett-Packard, Waldbronn, Germany) and compared to a gallic acid solution (3 g/L) used as standard. TPCs were calculated using the following formula: TPCs = 3 × (sample absorbance-blank absorbance)/(standard absorbance-blank absorbance). BHT was used as positive control. The results were expressed as mg gallic acid equivalents (GAE)/g sample. Each assay was carried out in triplicates.

### 3.7. DPPH Free-Radical-Scavenging Assay

The antioxidant activity of garlic, onion, leek, Chinese chive, shallot and chive essential oils was measured in terms of hydrogen-donating or radical scavenging ability by bleaching of purple colored methanolic solution of the stable radical DPPH [47]. The diluted solutions were prepared in methanol to obtain final concentrations ranging from 20 to 1 mg/mL of the stock essential oil solutions (20 mg/mL) and were introduced into test tubes.

Two milliliters of fresh methanolic solution of DPPH at a concentration of 6 × 10^−5^ M were added. BHT (0.1 mg/mL) prepared in diluted methanolic concentrations ranging from 0.01 to 0.08 mg/mL was used as positive control. The samples were shaken in the dark for four hours.

The decrease in absorbance at 517 nm was determined using a Hewlett-Packard 8453 diode-array spectrophotometer for all samples. Methanol was used to zero the spectrophotometer; Methanol and DPPH were used as negative control. All the samples were tested in triplicates.

The concentration of the sample required to decrease the absorbance of DPPH by 50% (IC_50_) was calculated graphically. The inhibition percentage of the DPPH radical was calculated according to the formula of Yen and Duh [48]:

I = (A_0_ − A_i_)/A_0_ × 100 (1)

where I = DPPH inhibition (%), A_0_ = absorbance of control sample (No antioxidant) t = 0, and A_i_ = absorbance of the tested sample at the end of the reaction (*t* = 4 h).

### 3.8. Frying Oil Test

The antioxidant effect of essential oils under heating conditions was studied by the determination of Total Polar Materials (TPMs). The objective of this test consisted of studying whether the addition of any of the EOs would increase the shelflife of fried sunflower oil. Each of the six essential oils (0.25 g) was added separately to sunflower oils (250 g) and the samples were heated under domestic frying conditions, *i.e*., 180 ± 5 °C during several hours [49]. The temperature was monitored by a thermocouple (ATC-300) inserted directly into the domestic deep-fat electric fryers. All samples were evaluated before the first heating sessions and every 1 h of heating until oil discard using a cooking oil tester (Testo 270, Testo Sàrl, Forbach, France). The end of the heating assays was determined by the value of TPM, where a maximum value of 25% is tolerated in accordance with the French law (Article 3-3 of decree No 86-857 of 18/07/86). This maximal legal content of TPM in frying oils, including hydrolysis products (diglycerides, monoglycerides and free fatty acids) and a complex distribution of oxidation products encompassing polymers, is formed at temperatures below 180 °C (French law No 86-857)The TPM value, usually assessed in restaurants and the agrofood industry by fast commercial tests (mostly based on colorimetric readings), has proven to correlate well with values obtained by official standards [49].

### 3.9. Statistical Analysis

Means and standard deviations of the assays were calculated using conventional statistical methods. Each treatment was performed in three replicates. Statistical analysis (ANOVA) was applied to the data to determine differences (*p* < 0.05). Means differences were made by using Tukey’s HSD test. The statistical analysis was carried out using Statgraphics XV.I for windows.

## 4. Conclusions

There are many ongoing studies on the biological properties of essential oils for their possible use as alternatives to synthetic antioxidant such as BHT. This study evaluates the composition of the essential oils of the six *Allium* plants, their antimicrobial, antioxidant activities and their possible use in food processing against thermal effects.

All the essential oils showed antioxidant properties with different degrees of scavenging activity. Shallot and leek oils were the strongest antioxidants. In the antimicrobial activity tests, all the essential oils inhibited a good range of Gram-positive and Gram-negative bacteria, while garlic, onion and Chinese chive were amongst the strongest. These activities are mainly attributed to the presence of the sulfur compounds in their compositions. Moreover, the variability in the composition, structure and concentration of the different sulfides present in the essential oils, play an important role in the determination of their antimicrobial and antioxidant activities.

Some components such as allyl sulfide group were reported for their biological properties, but other active components have not been fully investigated. Thus, further studies are needed on the sulfur compounds to link the chemical content with particular functional properties. Based on the results of the antimicrobial and antioxidant tests, essential oils can be applied as natural alternatives to food synthetic preservatives. However, heating should be carefully considered when the EO are used in cooking for antioxidant protection.
